# Beyond the Barrier: Overcoming Ocular Antimicrobial Resistance Through AI and Novel Therapeutics

**DOI:** 10.3390/ijms27167442

**Published:** 2026-08-20

**Authors:** Ambily Vasudevan, Abitha Sivanantham, Arya Bhai Seema Sreekumar, Mokshath Jayakrishnan Dayita, Namita Logeshwaran, Sivapriya Sindhu Suresh, Sandhya Padmakumar, Aravind Madhavan, Pradeesh Babu, Bipin G. Nair, Geetha B. Kumar

**Affiliations:** Amrita School of Biotechnology, Amrita Vishwa Vidyapeetham, Kollam 690525, Kerala, India; ambilynv@am.amrita.edu (A.V.); am.ls.u3bit23003@am.students.amrita.edu (A.S.); am.ls.u3bit23106@am.students.amrita.edu (A.B.S.S.); am.ls.u3bit23129@am.students.amrita.edu (M.J.D.); am.ls.u3mib23049@am.students.amrita.edu (N.L.); am.ls.u3bit23142@am.students.amrita.edu (S.S.S.); sandhyap@am.amrita.edu (S.P.); aravindmi@am.amrita.edu (A.M.); pradeeshbabu@am.amrita.edu (P.B.); bipin@am.amrita.edu (B.G.N.)

**Keywords:** ocular infections, antimicrobial resistance, ophthalmology, biofilms, microbial keratitis, artificial intelligence, current therapeutics

## Abstract

Ocular infections are a major cause of morbidity and vision loss worldwide, significantly affecting the quality of life and clinical outcomes. The management of ocular infections has become increasingly difficult due to the rising prevalence of antimicrobial resistance among commonly implicated pathogens. This review summarizes the epidemiology and etiology of ocular infections, with emphasis on bacterial pathogens frequently associated with resistance. Various mechanisms of antimicrobial resistance, including genetic mutations, intrinsic resistance, and biofilm formation, are also discussed. The review further examines the limitations of current therapeutics, such as poor ocular drug penetration, frequent dosing requirements, adverse effects, and reduced efficacy against multi-resistant organisms. In response to these challenges, the need for novel therapeutic approaches with improved stability and prolonged ocular retention is highlighted. Furthermore, the integration of artificial intelligence in ophthalmology is explored, particularly in disease diagnosis, image analysis, treatment planning, and antimicrobial resistance surveillance. Despite these advances, several translational challenges remain, including data set bias, limited external validation, regulatory approval hurdles, data privacy concerns, and restricted accessibility in resource-limited settings, which currently limit the widespread clinical implementation. Therefore, continued surveillance, rational antimicrobial use, and effective therapeutic strategies are essential to reduce the burden of ocular infections and improve patient outcomes.

## 1. Introduction

Ocular infections represent a significant global health burden, affecting multiple anatomical structures of the eye and posing a persistent threat to clear vision [1,2]. These pathologies arise from a diverse range of etiological agents, including viruses, fungi, and parasites; however, bacteria remain the primary cause of concern, accounting for approximately 50–70% of all clinical cases [1,3]. These infections commonly manifest as conjunctivitis, keratitis, endophthalmitis, and blepharitis [2]. If left untreated or managed with inadequate therapeutic regimens, bacterial pathogens can rapidly degrade delicate ocular tissues. The clinical consequences are often severe, ranging from corneal scarring and perforation to surgical flap failure and permanent sight loss [4]. In hyper-acute conditions such as endophthalmitis, extensive intraocular damage can occur within a critical 24–48 h window.

Among these conditions, conjunctivitis remains the most prevalent, followed by keratitis, exogenous endophthalmitis, blepharitis, and dacryocystitis [1,2]. Specifically, the principal causative agents of conjunctivitis include *Staphylococcus aureus*, *coagulase-negative staphylococci* (CoNS), *Streptococcus pneumoniae*, and *Haemophilus influenzae* [1,2], whereas *Pseudomonas aeruginosa* is the primary pathogen responsible for microbial keratitis [1,2]. Furthermore, *Corynebacterium* and *Cutibacterium acnes* are frequently associated with blepharitis, while *S. aureus* and *Streptococcus viridans* remain the leading causes of endophthalmitis (Table 1) [1,5]. Recent surveillance studies continue to identify bacterial pathogens as the predominant cause of culture-confirmed ocular infections worldwide. For example, the ARMOR surveillance program [6] and recent multicenter studies from Asia and Europe have reported that Gram-positive organisms, particularly staphylococcal species, account for more than 60% of bacterial ocular isolates, while *P. aeruginosa* remains the leading pathogen in contact lens-associated keratitis [7]. Furthermore, increasing resistance to fluoroquinolones and macrolides has been documented across multiple regions, highlighting the growing clinical burden of ocular AMR [8].

Beyond the virulence of the pathogens themselves, the eye’s natural defenses, such as high tear turnover, rapid nasolacrimal drainage, and the highly organized corneal epithelial barrier, function as a formidable shield, significantly limiting the drug penetration and reducing the time drugs remain in contact with the ocular surface [11]. Consequently, clinicians are frequently forced to use aggressive, high-frequency topical dosing or resort to more invasive and risky alternatives, such as systemic or intravitreal injections, to ensure an adequate quantity of medicine reaches the target tissue. This therapeutic struggle is now intensified by the escalating antimicrobial resistance (AMR) crisis [12]. In ophthalmology, increasing resistance trends in antibiotics such as macrolides, clindamycin, fluoroquinolones, and oxacillin are a major concern. Furthermore, the ability of these multidrug-resistant pathogens to form biofilms on contact lenses and intraocular devices provides a physical shield against both the host immune response and standard antibiotic therapy, leading to chronic, persistent infections [1,13,14].

In response, interdisciplinary approaches integrating artificial intelligence models assist in rapid diagnosis and treatment optimization, while nanomedicine-based delivery systems such as nanoparticles, liposomes, hydrogels, and other nanoformulations are improving ocular drug delivery through enhanced bioavailability, sustained release, and targeted therapeutic action with reduced toxicity. Bacteriophage therapy offers a promising alternative against multidrug-resistant pathogens and biofilms, whereas personalized medicine enables more precise and patient-specific treatment approaches. In addition, technologies such as organoids, organ-on-chip systems, and machine learning-based drug discovery platforms are advancing ophthalmic research and accelerating antimicrobial development [15,16]. Although the conventional ophthalmic formulations, including eye drops, ointments, and intravitreal injections, remain the fundamentals of ocular disease management, their therapeutic efficacy is often limited by poor corneal penetration, rapid tear clearance, frequent dosing requirements, and poor patient compliance. Nanotherapeutic platforms, including liposomes, polymeric nanoparticles, micelles, and hydrogels, have demonstrated improved ocular bioavailability, sustained drug release, enhanced tissue penetration, and targeted drug delivery. However, despite these advantages, several challenges continue to hinder their widespread clinical adoption [17]. The large-scale manufacturing of nanomedicines remains technically complex and costly, while concerns regarding long-term biocompatibility, formulation stability, reproducibility, and regulatory approval pathways remain largely unresolved. Furthermore, although numerous nanocarrier systems have demonstrated promising preclinical outcomes, relatively few have progressed to advanced clinical evaluation or routine ophthalmic practice. Therefore, while nanotherapeutics offer significant potential for overcoming current drug delivery limitations, further studies addressing safety, scalability, and clinical translation are required before widespread implementation can be achieved [17].

This review explores the integration of these diverse multidisciplinary approaches and highlights their potential to overcome the therapeutic challenges posed by the emerging AMR era in ophthalmology.

## 2. Epidemiology of Ocular Infections

The global landscape of ocular infections represents an escalating public health challenge, encompassing a diverse clinical spectrum that ranges from self-limiting conjunctivitis to sight-threatening microbial keratitis and endophthalmitis. In the contemporary era, the epidemiology of these pathologies is undergoing profound transitions driven by environmental shifts, socio-economic developments, and the persistent selective pressure of antimicrobial resistance (AMR). While Gram-positive bacteria typically predominate in aggregate global data, substantial regional specificities exist, most notably the rising incidence of Gram-negative pathogens and filamentous fungi in tropical zones [9,18].

In the United States, conjunctivitis accounts for approximately 1% of all primary care patient visits. The economic burden of the bacterial form alone is estimated between $377 million and $857 million annually [19]. While viral etiologies dominate globally (54–80%), South Asia is uniquely prone to explosive outbreaks [9]. A cross-sectional study from Babylon Governorate, Iraq, reported that 52.5% of patients with suspected external ocular infections showed positive bacterial cultures, with *Staphylococcus aureus* being the predominant pathogen and significant resistance observed against commonly used antibiotics such as ampicillin and penicillin [20].

Bacterial keratitis (BK) remains a primary cause of corneal blindness in India, accounting for nearly 44% of all infectious keratitis cases. In South India, Gram-negative isolates rose from 28% in 2013 to 41% in 2024 [21], with *Pseudomonas aeruginosa* predominating [20]. Conversely, Western countries report a continued dominance of Gram-positive *Staphylococci* (~60–70%) while Western isolates remain relatively stable in terms of emerging pathogens, South Asia has seen a post-pandemic surge in *Corynebacterium* spp. (rising from 4.2% to 16.1%), signaling a shift in the microbial landscape that challenges conventional empirical treatment [22]. In tropical and subtropical zones, fungal keratitis (FK) disproportionately impacts the population, accounting for 20% to 60% of all corneal infections. These infections are often triggered by agricultural trauma and are notoriously difficult to treat due to limited antifungal penetration and the slow-growing nature of the pathogens Table 1 [9,23].

In South Asia, the distinction between community and hospital-acquired resistance is blurring. Recent regional AMR evidence from the WHO South-East Asia Region further supports this concern. A 2023 Lancet Regional Health–Southeast Asia report noted that several countries in the region, including Bangladesh, India, Indonesia, Nepal, Sri Lanka, and Thailand, continue to show increasing drug resistance despite the implementation of national AMR action plans. The report also emphasized that fragmented surveillance systems, inappropriate antimicrobial use, weak regulatory enforcement, limited technical capacity, and financial constraints continue to hinder effective AMR control. In the context of ocular infections, these systemic challenges may partly explain the higher resistance burden observed in South Asia compared with Western settings, where antimicrobial stewardship programs, prescription regulation, and surveillance networks are generally more established. Therefore, regional differences in ocular AMR should not be interpreted only as microbiological variation, but also as a reflection of broader healthcare infrastructure, antibiotic accessibility, and surveillance capacity [24]. While community infections like acute conjunctivitis remain widespread, resistance to first-line fluoroquinolones (e.g., ciprofloxacin) in *S. aureus* has surged to 77%, a figure significantly higher than the 15–25% typically reported in North American community cohorts. In hospital settings, the crisis is more acute: South Asian surveillance indicates that 62.2% of post-surgical isolates exhibit multidrug-resistant (MDR) phenotypes, compared to approximately 30–35% in Western European facilities Table 1 [25].

Socio-economic factors and modern clinical trends complete this epidemiological picture by driving disease severity. In many regions, a median diagnostic delay of nearly two months is often due to low literacy or limited healthcare access, which allows minor infections to progress into vision-threatening conditions. This is frequently complicated by a widespread “steroid-first” culture, where over-the-counter misuse of steroid drops masks early symptoms while promoting deep-seated, recalcitrant diseases [26]. Ultimately, the landscape has evolved from acute, easily treated infections into a longitudinal trend of persistent, treatment-resistant cases that challenge standard clinical interventions.

## 3. Etiology and Pathogenesis of Ocular Infections

The eye is a highly specialized organ protected by a series of sophisticated anatomical and physiological defenses. The primary barrier is the corneal epithelium, a stratified layer of cells linked by tight junctions that prevents microbial ingress into the sterile stroma. This physical shield is augmented by the tear film, which contains potent antimicrobial proteins such as lysozyme, lactoferrin, and secretory IgA, and is continuously refreshed by the blink reflex. Under normal physiological conditions, these mechanisms effectively neutralize environmental pathogens [27]. However, when these defenses are compromised, most commonly through contact lens wear, mechanical trauma (particularly involving vegetative matter), ocular surface disease, or immunosuppression, the cornea becomes highly susceptible to rapid microbial colonization [28].

### 3.1. Pathogenesis of Infectious Keratitis (IK)

The transition from a healthy ocular surface to infectious keratitis follows a different pathological sequence. Once the epithelial barrier is disrupted, microorganisms attach to the exposed stromal collagen and begin to proliferate [29]. The specific etiology is often geographically dependent: while bacterial pathogens dominate in developed, temperate regions, fungal and filamentous organisms are more prevalent in tropical and agricultural settings [30].

### 3.2. Bacterial Infections

Bacterial ocular infections range from superficial mucosal colonization to rapid, sight-threatening intraocular destruction [10]. In conditions like conjunctivitis and blepharitis, pathogens such as *Haemophilus influenzae* and *Staphylococcus aureus* evade tear-film antimicrobials to trigger localized inflammation and discharge [10,31]. However, the most aggressive damage occurs in bacterial keratitis and endophthalmitis. *Pseudomonas aeruginosa*, a primary culprit in contact lens-related keratitis, utilizes specialized adhesion molecules and secretes a potent cocktail of proteases and exotoxins that directly liquefy the corneal stroma [31]. This invasion incites a massive “inflammatory storm” of neutrophils and cytokines; while intended to clear the infection, this overactive immune response often causes collateral tissue loss, leading to rapid corneal melting and perforation [32].

In the nutrient-rich, immune-privileged environment of the vitreous, endophthalmitis represents the most critical clinical emergency [10]. Gram-positive cocci and *Bacillus* species can proliferate unchecked, causing an explosive “vitritis” that leads to retinal detachment and photoreceptor death within 24–48 h [10,27]. Across all these pathologies, the clinical outcome is a direct result of the battle between bacterial virulence factors, such as *S. aureus* alpha-toxins or *P. aeruginosa* elastase, and the host’s inflammatory response. Without rapid intervention to disrupt this cycle of tissue degradation, these infections frequently result in permanent visual impairment or total globe failure Table 2 [27].

At the molecular level, bacterial pathogens are initially recognized by corneal epithelial cells and resident immune cells through pattern-recognition receptors (PRRs), particularly Toll-like receptors (TLRs). TLR2 recognizes Gram-positive bacterial components such as lipoteichoic acid and peptidoglycan, whereas TLR4 detects lipopolysaccharide (LPS) from Gram-negative bacteria, including *Pseudomonas aeruginosa*. Activation of these receptors initiates downstream MyD88-dependent signaling cascades, resulting in the activation of NF-κB and mitogen-activated protein kinase (MAPK) pathways. Consequently, infected epithelial cells produce a broad range of pro-inflammatory mediators, including IL-1β, IL-6, TNF-α, CXCL8 (IL-8), and other chemokines that orchestrate innate immune responses [33].

These inflammatory mediators promote the rapid recruitment of neutrophils to the infected cornea, which serve as the primary effector cells responsible for bacterial clearance through phagocytosis, degranulation, and reactive oxygen species (ROS) production [34]. Corneal epithelial cells further contribute to host defense through the secretion of antimicrobial peptides such as β-defensins and cathelicidins. Although these responses are essential for pathogen elimination, excessive or prolonged inflammation can become detrimental to host tissues [35].

During severe bacterial keratitis, activated neutrophils release proteolytic enzymes, elastases, myeloperoxidase, and matrix metalloproteinases (particularly MMP-2 and MMP-9), which degrade stromal collagen and extracellular matrix components. Simultaneously, elevated ROS production and persistent cytokine signaling amplify tissue injury, leading to corneal thinning, stromal melting, ulceration, and eventual perforation [36]. Therefore, ocular tissue damage results not only from direct bacterial virulence factors such as exotoxins and proteases but also from dysregulated host inflammatory responses. The balance between pathogen clearance and immune-mediated tissue injury ultimately determines disease severity and visual outcome [36].

### 3.3. Fungal and Parasitic Infections

Fungal and parasitic ocular infections present a more indolent but deeply invasive course compared to the rapid lysis seen in bacterial infections [1]. In the cornea, fungal keratitis caused predominantly by *Fusarium* and *Aspergillus*, which is characterized by hyphal extension deep into the stromal lamellae, often beyond the reach of topical fungal [1,2]. These infections typically manifest as “feathery” infiltrates and satellite lesions. Beyond the cornea, endogenous fungal endophthalmitis, often involving *Candida* species, can occur in immunocompromised patients, where yeast cells proliferate within the vitreous to form characteristic “string-of-pearls” or “fluff-ball” opacities that threaten the retina [2]. The management of fungal ocular infections is further complicated by the emergence of antifungal resistance and the ability of fungal pathogens to establish persistent biofilms. Resistance mechanisms in fungi include alterations in ergosterol biosynthesis pathways, mutations in antifungal target enzymes, overexpression of ATP-binding cassette (ABC) and major facilitator superfamily (MFS) efflux pumps, and activation of stress-response pathways that reduce susceptibility to azoles and polyenes. In addition, fungal biofilms formed by Candida, Fusarium, and Aspergillus species are embedded within an extracellular matrix that restricts antifungal penetration and promotes the survival of metabolically inactive cells, resulting in increased tolerance to treatment [37,38]. These biofilm-associated communities can persist on contact lenses, intraocular devices, and corneal surfaces, contributing to chronic and recurrent infections. Despite the availability of antifungal agents such as natamycin, amphotericin B, and voriconazole, therapeutic outcomes remain suboptimal due to poor corneal penetration, limited ocular bioavailability, prolonged treatment duration, local toxicity, and delayed diagnosis. Consequently, advanced fungal keratitis frequently requires surgical interventions, including therapeutic keratoplasty, to prevent progressive tissue destruction and vision loss [38].

Parasitic involvements, though less frequent, present unique therapeutic challenges due to their resilient life cycles [30]. *Acanthamoeba* is notorious for its ability to transition into a dormant cyst stage that resists both host immune responses and standard disinfectants, leading to chronic, ring-shaped corneal infiltrates and excruciating pain [4]. Across these diverse pathologies, the slow-growing nature and protective encystment of fungi and parasites often necessitate prolonged, multi-drug treatment regimens or surgical intervention Table 2 [27].

### 3.4. Viral Infections

Viral infections, predominantly driven by the Herpes Simplex Virus (HSV), initiate a complex pathological cycle that extends far beyond initial surface inflammation [27]. Recurrent viral activity often results in chronic stromal involvement and the development of neurotrophic keratopathy, a condition where the cornea loses its sensory innervation [4]. This desensitization critically impairs the blink reflex and stalls the natural wound-healing process, creating a state of permanent vulnerability [27]. Consequently, these compromised tissues frequently fall victim to secondary bacterial infections, which exploit the weakened epithelial barrier to accelerate tissue breakdown and deep stromal necrosis (Table 2).

**Figure 1:**
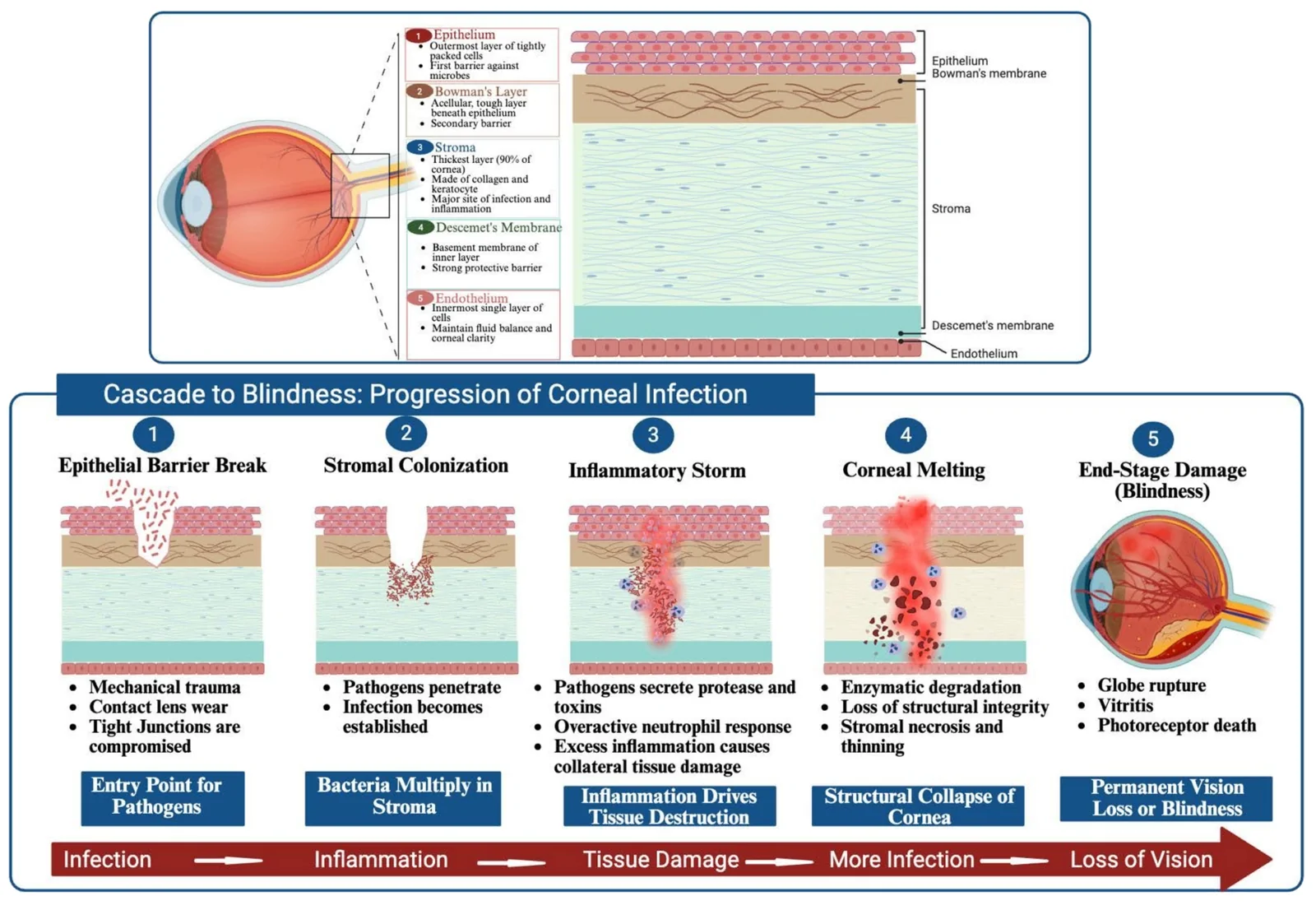
Corneal structure and cascade of blindness during progression of corneal infection. Visual representation of the anatomical layers of the cornea and the sequential progression of infectious keratitis leading to blindness.

Ultimately, the pathological sequence of all ocular infections, whether initiated by aggressive bacterial lysis, indolent fungal hyphae, or viral-induced neurotrophic decay converges toward a common, devastating end-stage. Regardless of the primary etiological agent or secondary complications like radiation-induced necrosis, the final trajectory involves a progressive loss of ocular structural integrity. Without rapid, targeted intervention to disrupt this cascade, the sequence moves inexorably from initial injury to stromal thinning and “corneal melting.” The culmination of this process is often globe rupture, endophthalmitis, or permanent visual loss, marking the complete failure of the eye’s biological and structural defenses Figure 1 [32].

## 4. AMR Mechanisms

Antimicrobial resistance (AMR) in ocular infectious pathogens is a multifactorial phenomenon involving a wide range of survival mechanisms that enable microorganisms to overcome antimicrobial therapy and persist within the ocular environment. These mechanisms extend from intrinsic resistance traits naturally present within microorganisms to acquired genetic adaptations and complex community-based survival strategies such as biofilm formation.

### 4.1. Intrinsic and Adaptive Mechanisms

Intrinsic resistance in ocular infections reflects baseline bacterial traits combined with unique anatomic and pharmacokinetic features of the eye [10]. *P. aeruginosa* keratitis isolates rely on chromosomally encoded efflux systems such as MexAB-OprM, MexXY-OprM and related pumps, together with low outer-membrane permeability, to limit intracellular accumulation of fluoroquinolones, β-lactams and aminoglycosides [10,39]. Overexpression of efflux pumps, including NorA and MepA, has also been reported in ocular *S. aureus* and CoNS exposed to topical fluoroquinolones and chloramphenicol, contributing to reduced susceptibility even when classical resistance genes are absent [10,39,40]. Loss or modification of porins such as OprD in Gram-negative corneal isolates further decreases uptake of carbapenems and related agents, driving high minimal inhibitory concentrations without acquired carbapenemases [41]. These bacterial attributes act in concert with host-derived barriers—rapid tear turnover, the multilayered corneal epithelium and glycocalyx, tight junctions in conjunctival and corneal epithelia, and limited penetration of many topical formulations to create steep antibiotic gradients and sub-inhibitory exposures on the ocular surface [40].

**Figure 2:**
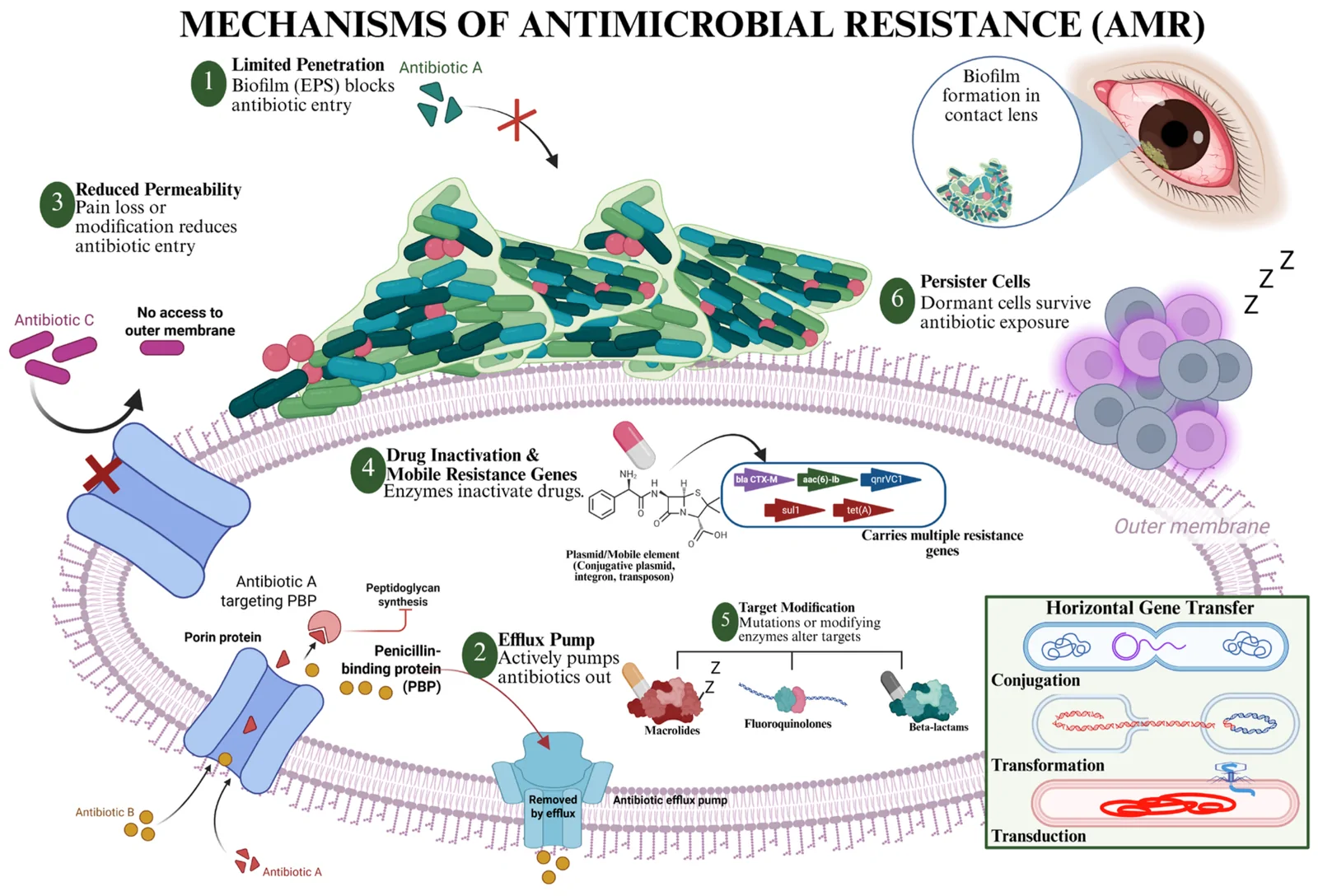
Mechanisms of Antimicrobial Resistance (AMR) in Ocular Pathogens. Schematic representation of major antimicrobial resistance mechanisms involved in ocular infections and biofilm-associated corneal disease. (1) Limited antibiotic penetration occurs due to the extracellular polymeric substance (EPS) matrix of biofilms, which restricts drug diffusion. (2) Efflux pumps actively expel antibiotics from bacterial cells, reducing intracellular drug concentration. (3) Reduced membrane permeability caused by altered porin proteins decreases antibiotic uptake. (4) Drug inactivation and acquisition of mobile resistance genes through plasmids, transposons, and integrons enable enzymatic degradation or modification of antibiotics. (5) Target modification through mutations or altered binding proteins reduces antibiotic efficacy against macrolides, fluoroquinolones, and β-lactams. (6) Persister cells survive antibiotic exposure in a dormant metabolic state, contributing to chronic and recurrent infections.

Beyond this static intrinsic resistance, *P. aeruginosa* and other pathogens exhibit adaptive resistance, a temporary survival response triggered by environmental stress, antibiotic exposure, or conditions within biofilms. Under these conditions, bacteria activate stress-response pathways, increase efflux pump activity, and alter gene expression to temporarily tolerate antimicrobial treatment without undergoing permanent genetic changes [41,42]. Distinguishing baseline intrinsic resistance from such adaptive, stress-induced tolerance is critical in ocular disease, because these transiently tolerant subpopulations can survive adequate topical regimens and later acquire stable mutations, especially within chronic biofilm-associated infections Figure 2 [41].

Importantly, adaptive resistance should be distinguished from genetically inherited resistance in the context of ocular infections. Adaptive resistance represents a transient and reversible physiological response that enables ocular pathogens to survive environmental stresses encountered on the ocular surface, including repeated exposure to topical antibiotics, host immune defenses, nutrient limitation, oxidative stress, and biofilm-associated growth on contact lenses, intraocular lenses, and corneal tissues. Under these conditions, bacteria can temporarily alter gene expression, increase efflux pump activity, reduce membrane permeability, and activate stress-response pathways, thereby decreasing antimicrobial susceptibility without acquiring permanent genetic changes [43]. This phenomenon contributes to the persistence and recurrence of infections such as microbial keratitis, conjunctivitis, and endophthalmitis, even when pathogens are initially susceptible to the administered antibiotic. Once the selective pressure is removed, bacterial susceptibility may be restored. In contrast, inherited resistance arises from stable genetic alterations, including chromosomal mutations or the acquisition of resistance determinants through plasmids, transposons, integrons, and other horizontal gene transfer mechanisms. These genetic changes are maintained during bacterial replication and can spread among bacterial populations, leading to persistent resistance phenotypes. Clinically, adaptive resistance often contributes to reduced treatment efficacy, biofilm persistence, and recurrent ocular infections, whereas inherited resistance drives the long-term emergence and dissemination of multidrug-resistant ocular pathogens, posing a significant challenge to the management of sight-threatening infections [18].

### 4.2. Genetic Mechanisms

Genetic mechanisms of antimicrobial resistance (AMR) in ocular pathogens are similar to systemic trends but are now well defined in corneal, conjunctival, and intraocular isolates (Table 3). Fluoroquinolone resistance in *Pseudomonas aeruginosa*, *Staphylococcus aureus* and *coagulase-negative staphylococci* (CoNS) from keratitis and conjunctivitis is driven by stepwise mutations in the quinolone-resistance-determining regions (QRDRs) of *gyrA/gyrB* and *parC/parE*, which reduce drug binding to DNA gyrase and topoisomerase IV [18,44]. Plasmid-mediated quinolone resistance (PMQR) genes such as *qnrVC1* in corneal *P. aeruginosa* isolates and *qnrA/qnrB/qnrS* in clinical *P. aeruginosa* more broadly provide target-protection proteins that shield DNA gyrase and topoisomerase IV, facilitating selection of high-level QRDR mutants under topical fluoroquinolone pressure [45].

In *Staphylococci* causing keratitis, methicillin resistance arises from acquisition of the *mecA* gene on SCCmec, encoding the low-affinity penicillin-binding protein PBP2a and conferring broad β-lactam resistance [18,44]. Macrolide resistance in ocular *S. aureus*, CoNS and streptococci is commonly mediated by erm-encoded ribosomal methyltransferases that modify the 23S rRNA target site and confer cross-resistance to macrolides, lincosamides and streptogramin B, while *mphC*, *msrA* and *mefA/msr* D contribute additional efflux-based macrolide resistance [44]. Gram-negative ocular isolates also increasingly harbor mobile β-lactamases, including ESBLs and carbapenemases, and aminoglycoside-modifying enzymes carried on plasmids, integrons and transposons, often clustering multiple resistance determinants in single elements [18,44].

Resistome studies of the conjunctival and corneal microbiota show a wide array of genes conferring resistance to macrolides, tetracyclines, trimethoprim-sulfamethoxazole and fluoroquinolones in both pathogens and commensals, creating a local gene pool that can be mobilized during infection [46]. Importantly, horizontal gene transfer (HGT) via conjugation and transformation is favored in dense, surface-attached communities, and biofilm models of *P. aeruginosa* demonstrate that the high cell density and extracellular DNA in the matrix enhance gene exchange, linking the surface-associated ocular biofilm lifestyle directly to the spread of resistance determinants on the eye Figure 2 [41].

## 5. Biofilm and Persistence

Biofilms are structured, surface-attached microbial communities embedded in a self-produced extracellular polymeric substance (EPS) matrix composed of polysaccharides, proteins, lipids and extracellular DNA, generating steep nutrient and oxygen gradients and a continuum of metabolic states from actively dividing cells to dormant persister cells [47]. In ocular settings, biofilm development begins with an early attachment phase in which staphylococci use MSCRAMMs (microbial surface components recognizing adhesive matrix molecules) to bind fibrinogen, fibronectin and other host proteins [47,48], while *P. aeruginosa* exploits flagella, type IV pili and outer membrane adhesins to attach to corneal epithelium and biomaterials [49]. Tear proteins such as lysozyme, lactoferrin and albumin rapidly form a “conditioning film” on contact lens surfaces, modifying hydrophobicity and providing host-matrix ligands that enhance bacterial adhesion and subsequent microcolony formation [41]. After initial attachment, organisms proliferate into microcolonies, produce EPS and mature into complex three-dimensional structures on external ocular surfaces (corneal epithelium and stroma in microbial keratitis, conjunctival sac and lid margin in chronic conjunctivitis/blepharitis, and soft and rigid contact lenses and their cases) and on internal devices (posterior capsule and intraocular lens [IOL] surfaces in delayed-onset endophthalmitis, sutures, scleral buckles and lacrimal drainage implants) [41,50]. Within these biofilms, Gram-positive organisms such as *S. aureus* and CoNS, Gram-negatives including *P. aeruginosa* and *Enterobacterales*, and fungi such as *Candida albicans* and *Fusarium* spp. coexist, and biofilm-embedded *P. aeruginosa* relies on quorum-sensing networks (las, rhl and PQS systems) to coordinate EPS production, virulence factor expression and dispersion in response to cell density and environmental cues [51,52]. Acanthamoeba trophozoites exploit pre-existing bacterial and fungal biofilms on lenses and cases as nutritional and protective niches, increasing the risk and severity of amoebic keratitis [52,53,54].

The clinical implications of ocular biofilms are substantial, as microorganisms within biofilms often demonstrate enhanced antimicrobial tolerance compared with planktonic cells. The degree of antimicrobial tolerance exhibited by biofilm-associated ocular pathogens varies considerably depending on the microbial species, antimicrobial agent, and stage of biofilm development. Early biofilms may remain partially susceptible to antimicrobial therapy; however, as the biofilm matures, the production of a dense extracellular polymeric matrix restricts antibiotic penetration and creates microenvironments that support slow-growing and metabolically inactive bacterial populations. In ocular infections, particularly those associated with contact lenses, intraocular lenses, corneal ulcers, and endophthalmitis, mature biofilms can significantly reduce the effectiveness of topical and systemic antimicrobial agents. Furthermore, the presence of persister cells and altered physiological states within mature biofilms enables bacterial survival despite prolonged antibiotic exposure, contributing to treatment failure, chronic infection, and disease recurrence. Consequently, established biofilm-associated ocular infections are substantially more difficult to eradicate than their planktonic counterparts and often require prolonged therapy or device removal in severe cases [47,50]. On contact lenses and their storage cases, biofilms of *P. aeruginosa*, *S. aureus*, CoNS, *Fusarium* spp. and *C. albicans* transform asymptomatic colonization into sight-threatening keratitis when aggregates and dispersed cells repeatedly adhere to the corneal surface during lens wear. This pathogenic process has also been demonstrated in experimental models where fungal biofilm-covered silicone hydrogel lenses reliably induce keratitis [41,50,52]. Biofilm on IOLs and capsular bags underlies low-grade, recurrent postoperative endophthalmitis caused by *Cutibacterium acnes*, CoNS and *Enterococcus faecalis*, while biofilm on sutures and scleral buckles sustains chronic conjunctive or scleral inflammation [55]. Management typically requires high-dose topical, intrastromal or intravitreal antibiotics (often fortified combinations targeting both Gram-positive and Gram-negative organisms), together with mechanical or surgical measures such as corneal debridement, intensive lens and case replacement protocols, suture removal, or IOL and capsular bag explantation for recalcitrant intraocular infections Figure 2 [50].

### Biofilms and Chronic Infections

Biofilms are tightly linked to chronic and recurrent ocular infections, shaping disease phenotype and outcome in contact-lens-associated microbial keratitis, infectious crystalline keratopathy, chronic blepharitis and meibomian gland dysfunction, fungal keratitis, Acanthamoeba keratitis and delayed onset postoperative endophthalmitis [51,54,55]. In contact-lens–related microbial keratitis, mature bacterial and fungal biofilms on lenses and cases serve as persistent reservoirs from which planktonic cells and aggregates are repeatedly shed onto the corneal epithelium. These pathogens exploit microtrauma and hypoxia induced by prolonged lens wear to establish ulcers that often progress to deep stromal involvement, melting and scarring. In many cases, infections may persist or recur even when surface cultures become temporarily negative, as the biofilm reservoir on the lens or storage case remains unaffected [29,41,51,55].

Infectious crystalline keratopathy represents a slow-progressing stromal biofilm disease in which sparsely distributed microcolonies of viridans streptococci or CoNS embed within the corneal stroma, producing needle-like crystalline opacities. They exhibit minimal inflammation and a poor response to short antibiotic courses, which leads to frequent relapse unless very prolonged or targeted therapy is used [50]. Similarly, biofilms formed by staphylococci along the eyelid margin and ocular surface can produce toxins and lipases that disrupt the tear film and promote chronic low-grade inflammation. This contributes to persistent blepharitis, meibomian gland dysfunction, worsening dry eye symptoms, and increased susceptibility to secondary microbial infections [56].

Fungal biofilms of *Fusarium* and *C. albicans* on contact lenses cause central to severe, refractory fungal keratitis, where hyphal penetration, robust EPS and deep stromal invasion combine with limited penetration and fungicidal activity of topical azoles or polyenes, leading to prolonged courses, frequent therapeutic keratoplasty and suboptimal visual outcomes [29,54]. Acanthamoeba keratitis illustrates how protozoa exploit mixed biofilms: Acanthamoeba trophozoites preferentially colonize bacterial biofilms, particularly those containing *P. aeruginosa*, on lens surfaces and cases, gaining protection from disinfectants and host defenses and contributing to highly painful, chronic keratitis that is notoriously difficult to eradicate [29,41,51,55].

Inside the eye, delayed-onset endophthalmitis, which is manifested months after cataract surgery, is caused by organisms such as *C. acnes*, CoNS and *E. faecalis* that persist in biofilms on the posterior IOL surface or within the capsular bag. Intravitreal antibiotic therapy may provide temporary suppression of infection; however, complete eradication of biofilm-associated microorganisms is often challenging once mature biofilms become established on intraocular lenses or within the capsular bag. In selected cases of persistent or recurrent postoperative endophthalmitis, durable infection control may require intraocular lens explantation and capsulectomy, which are invasive surgical interventions performed when antimicrobial therapy alone is insufficient to eliminate the underlying biofilm-associated infection [55]. Across these entities, the presence of metabolically quiescent persister cells within ocular biofilms can survive very high antibiotic concentrations and can repopulate the biofilm once therapy is reduced. This will mechanistically explain the chronicity, recurrence and vision-threatening outcomes that position biofilm biology at the center of AMR and disease persistence in ophthalmology [47,57].

## 6. Current Therapeutic Strategies and Challenges

The clinical management of ocular infections necessitates a rapid, tiered approach where the primary objective is to arrest microbial proliferation before irreversible structural damage occurs [58,59]. This process typically begins with empirical therapy, utilizing broad-spectrum antibiotics to provide immediate coverage against a wide array of potential pathogens [59,60]. While this early intervention is critical for preventing rapid corneal melting, it carries the inherent risk of inducing bacterial stress responses or failing to address specific resistant phenotypes that have not yet been identified [61]. Consequently, clinicians aim to transition toward culture-guided therapy as soon as laboratory results are available [59,62]. This shift allows for a more precise, narrow-spectrum hit that maximizes efficacy while reducing the risks of systemic toxicity and the unnecessary selection of multidrug-resistant strains [59].

Topical administration remains the gold standard for treating anterior segment infections, such as conjunctivitis and keratitis, as it provides high local drug concentrations with minimal systemic side effects [58,63]. However, deeper or more aggressive infections, such as endophthalmitis, often require more invasive delivery routes, including systemic or intravitreal injections [58,64]. To support these antimicrobial efforts, several adjunctive therapies are integrated into the treatment plan to preserve ocular integrity. For instance, cyanoacrylate glue serves as a vital structural sealant for corneal thinning or small perforations, while amniotic membrane transplantation (AMT) acts as a bioactive scaffold that promotes epithelial healing through its unique anti-inflammatory, anti-fibrotic, and anti-angiogenic properties [10,65].

Furthermore, autologous serum eye drops and conjunctival flap surgery provide essential growth factors and physical protection for severe, non-healing ulcers, although these procedures must be balanced against the risk of long-term visual impairment. Beyond standard care, other experimental therapies have been explored with varying degrees of success; povidone-iodine offers broad-spectrum antiseptic activity but lacks deep stromal penetration. Cryotherapy has shown isolated benefits in severe, antibiotic-resistant Pseudomonas keratitis [58,66]. Additionally, antimetabolites like mitomycin-C and matrix metalloproteinase inhibitors are being studied to reduce scarring and tissue degradation, though strong clinical evidence for their routine use remains limited [58,65,67].

Conventional treatment of ocular infections relies on a range of topical, systemic, and intravitreal antimicrobial agents. Commonly prescribed antibiotics include fluoroquinolones such as moxifloxacin (0.5% ophthalmic solution) and gatifloxacin (0.3%), aminoglycosides such as tobramycin (0.3%), and fortified antibiotics including vancomycin (25–50 mg/mL) and ceftazidime (50 mg/mL) for severe bacterial keratitis. Antifungal therapy typically involves natamycin 5% suspension, amphotericin B (0.15–0.3%), or voriconazole 1% eye drops, while antiviral infections are commonly treated with topical ganciclovir 0.15% gel or oral acyclovir. Treatment duration may range from several days to weeks, depending on the severity and etiology of the infection, often requiring frequent administration, particularly during the acute phase [29]. Despite their effectiveness, these therapies are associated with several limitations, including poor ocular bioavailability, limited corneal penetration, and patient non-compliance resulting from frequent dosing schedules. In addition, adverse effects such as ocular irritation, burning sensation, conjunctival hyperemia, allergic reactions, corneal epithelial toxicity, and blurred vision may compromise treatment adherence. Prolonged or inappropriate antimicrobial use can further promote the emergence of resistant pathogens, thereby reducing therapeutic efficacy and contributing to the growing burden of antimicrobial resistance in ocular infections [68].

However, several fundamental limitations continue to hinder optimal patient outcomes, most notably the eye’s own pharmacokinetic barriers. The continuous production of tears and the highly organized corneal epithelium act as a biological shield that rapidly clears medications, necessitating a grueling schedule of frequent dosing. This intensive regimen often leads to ocular surface toxicity and poor patient compliance, which in turn creates sub-therapeutic drug gradients that allow infections to persist [63,69]. Furthermore, the effectiveness of standard antibiotics is increasingly compromised by the formation of biofilms on both biotic corneal tissue and abiotic surfaces like contact lenses or intraocular lenses [13,70]. Within these structured microbial communities, bacteria are embedded in a protective extracellular polymeric substance (EPS) matrix that reduces antibiotic penetration by up to 1000-fold and shields metabolically inactive “persister” cells from the immune system [13,71,72]. The presence of these persister cells is a primary driver of treatment failure and clinical relapse, as they remain unaffected by drugs that target active cell division and can repopulate the infection once therapy is discontinued. This challenge is particularly acute in contact lens-related keratitis, where contaminated lenses and storage cases serve as ongoing reservoirs for pathogens like *Pseudomonas aeruginosa* [73,74,75]. Ultimately, the synergy between rising antimicrobial resistance, biofilm-mediated protection, and the physical limitations of drug delivery highlights a critical gap in current ophthalmic practice. Addressing these bottlenecks requires a shift toward more advanced analytical tools, such as AI-based research, to predict resistance patterns and guide the development of innovative, anti-biofilm therapies like bacteriophages or enzyme-based EPS degraders that can penetrate the deeper layers of the ocular stroma [64,70].

Antifungal medications are very important in treating fungal keratitis. In many cases, doctors use topical antifungal eye drops such as voriconazole and natamycin [75]. Voriconazole was used in about 71.4% of patients, while natamycin was used in around 53.6% of cases. Natamycin is usually considered the first choice of treatment, especially for infections caused by filamentous fungi. Along with eye drops, oral voriconazole was given to about 64.3% of patients to help the medicine reach deeper layers of the eye and improve the effectiveness of treatment [76,77]. However, treating fungal keratitis can be challenging because antifungal drugs do not easily reach the deeper part of the cornea. In addition, the disease is sometimes diagnosed late since fungal keratitis can look similar to bacterial keratitis, and fungal cultures take more time to grow and confirm the infection [76]. Because of these difficulties, surgery was required in about 32.1% of cases, including procedures such as therapeutic penetrating keratoplasty, which is a type of corneal transplant. Since filamentous fungi were the most common cause of infection in the study, the use of voriconazole was particularly highlighted. Other adjuvant methodologies like Rose Bengal photodynamic therapy can be used for treating highly infectious keratitis [78]. Overall, the findings suggest that using both topical and oral antifungal medicines together, along with careful monitoring for complications such as corneal thinning, perforation, and secondary bacterial infections, is important for effective management [79].

Current therapeutic strategies for viral infections of the ocular surface vary by pathogen [80]. For Herpes Simplex Virus (HSV) keratitis, topical or oral antivirals like acyclovir and valacyclovir form the mainstay, with oral prophylaxis reducing recurrences; corticosteroids manage stromal keratitis and endotheliitis alongside antivirals [81,82], while emerging options include recombinant human nerve growth factor (rhNGF) for neurotrophic keratopathy, sustained-release nanocarriers, and preclinical HSV vaccines targeting glycoproteins and latency genes [82]. Adenoviral conjunctivitis (EKC) relies on supportive care due to no FDA-approved antivirals, cautious topical corticosteroids for subepithelial infiltrates, adjunctive 5% povidone-iodine, and investigational agents like sialic acid conjugates, GAG mimetics, cold atmospheric plasma, and CRISPR-Cas9 [80]. Cytomegalovirus (CMV) keratitis uses PCR-confirmed diagnosis from aqueous humor, topical ganciclovir or oral valganciclovir for treatment, next-generation antivirals (letermovir, maribavir) for resistance, adoptive T-cell immunotherapy, and metagenomic next-generation sequencing (mNGS) for monitoring [82,83]. Treatment of HPV-related ocular surface squamous neoplasia (OSSN) usually involves surgical removal of the lesion along with cryotherapy to prevent recurrence. Eye drops such as interferon-alpha 2b, mitomycin-C, or 5-fluorouracil are also commonly used. HPV vaccination against high-risk types (16, 18, and 33) may help in prevention. In advanced cases, immune-based therapies like checkpoint inhibitors can be used. Detection of HPV is done using RNA in situ hybridization to identify E6/E7 genes. Broader emerging strategies encompass immunomodulators like topical cyclosporine and tacrolimus, nanomedicine innovations (liposomes, in situ gels), the recombinant zoster vaccine as a prevention model, and AI for diagnostics and surveillance Table 4 [84].

## 7. Emerging and Adjunctive Therapies

The continuous emergence of novel infectious agents, along with the rapid evolution of antimicrobial resistance (AMR), has created a major global healthcare challenge that increasingly limits the effectiveness of conventional therapeutics. Pathogens are developing sophisticated survival strategies, including biofilm formation, intracellular persistence, horizontal transfer of resistance genes, immune evasion, and adaptation to hostile microenvironments, enabling them to withstand both host defenses and antimicrobial interventions. At the same time, the emergence of multidrug-resistant, extensively drug-resistant, and even pan-drug-resistant strains has significantly reduced the efficacy of existing antibiotics and antifungal agents. Consequently, increasing attention is being directed toward advanced and emerging treatment modalities, particularly nanomedicine and bacteriophage therapy, which offer promising alternatives for overcoming resistance, enhancing targeted drug delivery, disrupting biofilms, and improving infection control against evolving pathogens [85,86].

### 7.1. Nanomedicine

Nanomedicine encompasses a wide array of nanomaterials, including liposomes, exosomes, and metal-based nanoparticles such as gold and silver. Among lipid-based nanoparticles, liposomes are spherical vesicles composed of phospholipids that exhibit several advantageous properties, including protection of drugs from physiological degradation and prolonged circulation time. However, their application as adjunctive therapies in ocular infections remains under investigation [85]. Metal nanoparticles demonstrate antimicrobial efficacy through multiple mechanisms. Silver nanoparticles have been widely studied for their antibacterial activity against pathogens associated with bacterial keratitis. Gold particles, on the other hand, possess anti-angiogenic and anti-inflammatory properties that may provide additional advantages for ocular drug delivery [85,87].

In addition to lipid-based nanocarriers, several polymer-based drug delivery systems have emerged as promising alternatives for enhancing ocular drug administration. Polymeric nanoparticles, polyelectrolyte nanoparticles, polymeric micelles, dendrimers, and chitosan-based nanocarriers have attracted considerable attention due to their excellent biocompatibility, biodegradability, and ability to improve drug pharmacokinetics. These systems enhance mucoadhesion and prolong precorneal residence time, thereby reducing rapid drug clearance caused by tear turnover and blinking. Furthermore, they protect encapsulated drugs from degradation, improve drug solubility and stability, and enable controlled and sustained drug release, resulting in prolonged therapeutic effects and reduced dosing frequency. Chitosan-based carriers, in particular, can transiently open tight junctions and facilitate enhanced corneal penetration, while polymeric micelles are especially effective for delivering poorly water-soluble drugs. Additionally, polyelectrolyte nanoparticles offer high drug-loading capacity and tunable release profiles through electrostatic interactions. Collectively, these polymer-based nanocarriers have demonstrated significant potential for improving ocular bioavailability, enhancing therapeutic efficacy, minimizing systemic exposure, and overcoming the limitations associated with conventional ophthalmic formulations [88,89].

Nano-emulsions are another class of drug delivery vehicles characterized by high kinetic stability and resistance to changes in temperature and dilution, enabling efficient delivery of therapeutic agents to intraocular tissues. For instance, a nano-emulsion formulation (MM3) combined with moxifloxacin achieved greater than 95% transmittance, which indicates excellent ocular clarity while delivering drug concentration in the aqueous humor above threshold concentration [90].

In fungal keratitis, phenylboronic acid conjugated chitosan oligosaccharide vitamin E copolymer (PBA-CS-VE) nanoparticles loaded with voriconazole have demonstrated prolonged ocular retention. This effect is attributed to the specific interactions between phenylboronic acid and sialic residues in ocular mucins, which form high-affinity complexes, increasing the drug residence time [91].

### 7.2. Bacteriophage Therapy

Besides the advances in material-based therapeutics, phages as an intervention for infections that cannot be treated by antibiotics have re-emerged as an alternative strategy. Bacteriophages preserve the commensal microbiota while selectively lysing bacterial pathogens. This becomes especially valuable in the management of sight-threatening infections caused by MDR pathogens such as *Pseudomonas aeruginosa*, *Staphylococcus aureus*, and *Escherichia coli*. In addition to the targeted action, phages also disrupt biofilms and undergo in situ replication, enabling sustained antimicrobial activity [86]. Building on this potential, recent in vitro studies have demonstrated the broad activity of bacteriophages against multidrug-resistant ocular pathogens. Investigations involving ocular *Staphylococcus aureus* isolates, including MRSA strains, have shown that a majority of isolates are susceptible to at least one phage, with strong dose-dependent bactericidal effects. These findings reinforce the growing role of phage-based therapeutics as alternative strategies for managing antimicrobial-resistant ocular infections [92]. Further advancements, such as the development of nano-helmets that encapsulate bacteriophages, enhance ocular retention time. The combination of phages and nanomaterials enhances the antimicrobial efficacy while protecting phages [92]. Beyond bacteriophage therapy, enzyme-based approaches have emerged as promising alternatives for the treatment of ocular infections. Bacteriophage-derived endolysins are highly specific peptidoglycan hydrolases capable of degrading bacterial cell walls and inducing rapid bacterial lysis, including activity against antibiotic-resistant pathogens. Similarly, bacteriolytic enzymes such as lysozyme, a natural component of the tear film and innate immune defense system, can hydrolyze bacterial peptidoglycan and contribute to pathogen clearance. These enzymes have also demonstrated the ability to disrupt microbial biofilms, thereby enhancing antimicrobial penetration and improving therapeutic efficacy against persistent infections [93].

In addition to their antimicrobial activity, enzymes with anti-inflammatory and antioxidant properties have attracted increasing interest. Superoxide dismutase (SOD), a key antioxidant enzyme, catalyzes the conversion of superoxide radicals into less reactive molecules, thereby reducing oxidative stress and limiting inflammation-induced tissue damage. Such enzymes may help preserve ocular tissue integrity, promote wound healing, and improve clinical outcomes when used alone or in combination with conventional antimicrobial therapies. Collectively, enzyme-based therapeutics represent a promising adjunctive strategy for addressing antimicrobial resistance while simultaneously controlling infection-associated inflammation [94].

Despite these promising developments, several challenges limit the routine clinical application of phage therapy. The high specificity of phages requires rapid and accurate identification of the causative pathogen, which may be time- intensive in clinical settings. Additionally, bacteria can develop resistance to phages through mechanisms such as receptor modification or activation of defense systems like CRISPR–Cas. Overcoming these limitations will require the development of well-characterized phage libraries alongside faster diagnostic tools. Moreover, combining phage therapy with conventional antibiotics or engineering phages with enhanced activity may further improve treatment outcomes and reduce resistance [86].

## 8. AI in Ophthalmology

Artificial intelligence (AI) is increasingly being integrated into ophthalmology due to its ability to analyze large volumes of clinical and imaging data with high speed and accuracy. Advances in machine learning and deep learning have expanded the application of AI across multiple areas of ophthalmic care, improving clinical decision-making and healthcare accessibility. AI-based technologies are now being explored for ocular disease diagnosis, therapeutics, prophylaxis, and surveillance to enable earlier detection, targeted interventions, and efficient disease monitoring.

### 8.1. Diagnosis

**Figure 3:**
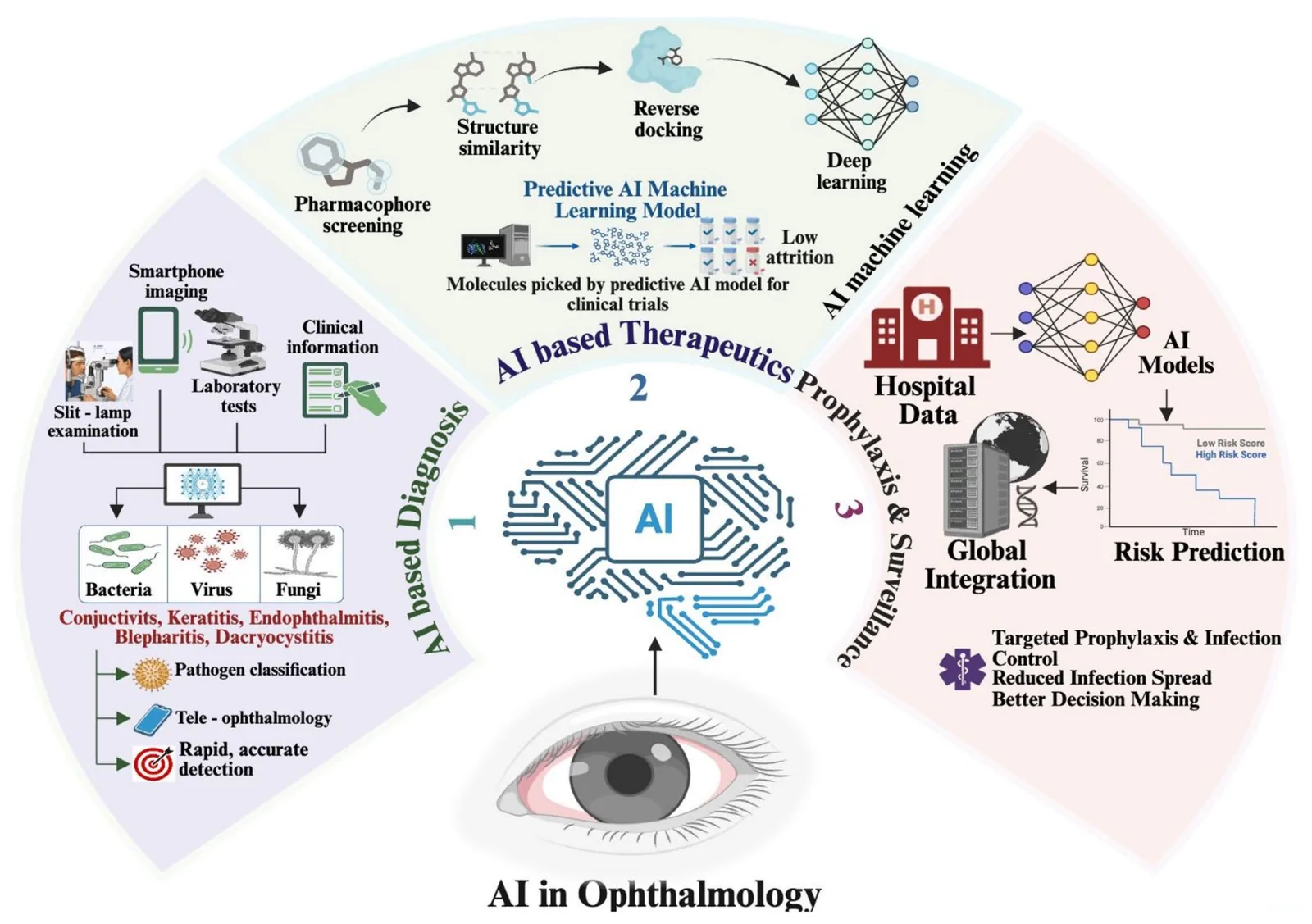
Applications of artificial intelligence in ophthalmology created in www.Biorender.com. Schematic representation of the major roles of AI in ophthalmology, including (1) AI-based diagnosis, which integrates data from slit-lamp imaging, smartphone-based imaging, laboratory tests, and clinical records to enable rapid detection and classification of ocular pathogens such as bacteria, viruses, and fungi, supporting conditions like conjunctivitis, keratitis, endophthalmitis, blepharitis, and dacryocystitis. (2) AI-driven therapeutics, pharmacophore screening, structure similarity analysis, reverse docking, and deep learning models facilitate drug discovery and optimization, improving clinical trial efficiency and reducing attrition rates. (3) prophylaxis and surveillance, AI models utilize hospital and global datasets to predict infection risk, guide targeted prophylaxis strategies, enhance infection control, and support clinical decision-making.

Vision-threatening ocular infections require timely and accurate identification of the causative pathogen. But the current gold standard for diagnostics has inherent limitations such as low microbial culture yield and prolonged turnaround times [95]. Advances in machine learning and artificial intelligence have enabled the development of AI-driven models capable of accurately differentiating the etiologies of common ocular infectious diseases, such as keratitis, particularly in distinguishing between fungal and bacterial infections [96]. Deep learning models, in some cases, have surpassed general ophthalmologists and approached the levels of corneal specialists but fungal keratitis remains challenging to differentiate with consistent accuracy [97]. Classification outcomes can be influenced not only by algorithm design but also by factors such as image brightness and illumination conditions. AI has also shown its ability to analyze images that were captured through a slit-lamp as well as a smartphone-based system. In resource-limited settings, the use of portable models supports the feasibility of telemedicine applications, as it minimizes memory requirements and eliminates the need for manually annotated images Figure 3 [98].

### 8.2. Therapeutics

Machine learning models such as quantitative structure–activity relationship (QSAR) play an important role in identifying drug-target interaction, which enables rational drug design and lead optimization [16]. Peptide-based models can help predict how antimicrobial peptides work, and some have shown strong results in living systems, making them useful for speeding up drug discovery [99]. Generative AI models can also design new and diverse peptide-based drugs. To study drug safety and behavior before clinical trials, organoids and organ-on-chip systems are being used as alternatives to animal models. Organoids are 3D structures grown from stem cells that resemble real organs, while organ-on-chip devices are small platforms that mimic how organs function. Using similar approaches with eye (ocular) organoids could improve the accuracy of eye treatments [100].

AI models can also predict how drugs move through the eye by combining chemical properties of the drug with imaging and anatomical data, helping to estimate drug distribution and pharmacokinetics [101]. In addition, clinical trials can be monitored using deep learning models connected to smartphones, allowing patients to participate more easily. Overall, these AI-based approaches can help reduce the time needed for drug discovery and development Figure 3 [16,98].

Despite their considerable promise, several challenges continue to limit the clinical translation of AI-assisted therapeutics and anti-biofilm strategies. AI-based diagnostic and treatment platforms require large, high-quality datasets for training and validation, while concerns regarding data privacy, algorithm transparency, regulatory approval, and integration into existing clinical workflows remain unresolved. Similarly, although anti-biofilm approaches such as bacteriophage therapy, enzyme-based biofilm disruptors, and nanotherapeutics have demonstrated encouraging pre-clinical results, their widespread clinical adoption is hindered by manufacturing complexity, formulation stability, large-scale production challenges, regulatory requirements, and limited long-term safety data. Consequently, further standardization, clinical validation, and regulatory assessment are required before these emerging technologies can be routinely incorporated into ophthalmic practice [15].

### 8.3. Prophylaxis and Surveillance

A more proactive and data-driven approach to infection prevention and control can reduce the burden of antimicrobial resistance. In this context, machine learning models that predict surgical site infections using preoperative risk factors enable targeted prophylactic interventions [102]. Through analysis of hospital-acquired clinical data, Conventional treatment of ocular infections can predict the spread of infection within healthcare facilities. These predictive frameworks can limit the transmission of resistant pathogens. Predictive modeling of resistance patterns and novel resistant determinants can be facilitated using AI. In healthcare settings that are resource-constrained, the integration of genomic, epidemiologic and environmental data can enable rapid AI-driven analysis of resistance trends [103]. Furthermore, integration with global surveillance frameworks such as the WHO Global Antimicrobial Resistance and Use Surveillance System (GLASS) enhances cross-border monitoring and coordinated response strategies [15].

Despite their promising diagnostic performance, AI-based systems face several challenges, including dataset bias, limited external validation across diverse populations, and the lack of explainable decision-making processes. Furthermore, regulatory approval requirements, data privacy concerns, and integration into clinical workflows remain important barriers to widespread clinical adoption. Given the distinct microbiological profiles, with the localized nature of infection and constraints in ophthalmic datasets, there exists a significant research gap in translating these approaches to improve prevention and antimicrobial management in ocular infections Figure 3.

## 9. Conclusions and Future Perspectives

Ocular infections continue to impose a substantial global health burden, affecting multiple eye structures and threatening clear vision across diverse ages and populations. Although viral, fungal and parasite pathogens also contribute, bacterial infections are the most common, contributing to about 50–70% cases through conjunctivitis, keratitis, endophthalmitis, and blepharitis. Because the eye is a delicate and immune-privileged organ, delayed diagnosis or inadequate treatment can lead to inflammation, tissue damage, visual impairment or even irreversible blindness. Conventional treatments for ocular infections falter due to multiple reasons. Poor ocular bioavailability of topical medications, frequent dosing requirements and multidrug resistance to antibiotics, along with biofilm-associated persistence, make conventional methods less reliable and prompt us to shift our focus to novel therapies for more effective, targeted and sustained solutions.

The rising burden of antimicrobial resistance (AMR) in ocular infections is driving the need for more precise and adaptive therapeutic strategies. Artificial intelligence (AI) is expected to play a significant role in transforming the diagnosis and management of ocular infections through rapid, accurate, and personalized clinical decision-making. AI-integrated imaging systems and microbiological databases may enable early differentiation of bacterial, fungal, viral, and parasitic infections while assisting clinicians in predicting antimicrobial resistance patterns and optimizing treatment strategies. Simultaneously, advances in nanomedicine, including nanoparticle-based drug delivery systems such as liposomes, hydrogels, dendrimers, and nanoemulsions, may improve corneal penetration, prolong drug retention, and provide sustained therapeutic release, thereby overcoming the limitations of conventional topical therapies and enhancing treatment efficacy.

Parallelly, phage therapy is emerging as a promising alternative or adjunctive approach for multidrug-resistant ocular infections due to its host specificity, biofilm-disrupting capability, and self-replicating nature. Future developments involving engineered phages, phage-derived enzymes, and combination therapies with antibiotics or nanocarriers may further improve antimicrobial effectiveness while reducing resistance development. In addition, other evolving technologies such as antimicrobial peptides, CRISPR-Cas-based therapeutics, photodynamic therapy, probiotics, and immunomodulatory approaches may broaden future treatment options. Advanced experimental platforms, including organoids, tissue-engineered corneal models, and organ-on-chip systems, are also expected to accelerate translational research and improve the evaluation of novel ocular therapeutics. In conclusion, the management of ocular infections is moving beyond conventional empirical therapies toward more targeted and advanced approaches involving computational biology, engineered therapeutics, and precision medicine. The future management of ocular infections is rapidly advancing toward a highly integrated, personalized, and precision-based strategy that continuously evolves to meet the growing challenges posed by the antimicrobial resistance (AMR) era.

Despite the significant potential of AI-assisted diagnostics and emerging therapeutic platforms, several challenges must be addressed before their widespread clinical adoption. Ethical concerns related to data privacy, algorithm transparency, and clinical accountability remain important considerations. Furthermore, the high cost of advanced technologies, regulatory requirements, and limited healthcare infrastructure may restrict implementation, particularly in resource-limited settings. Therefore, future efforts should focus not only on technological innovation but also on improving accessibility, affordability, and clinical validation to ensure equitable healthcare delivery.

## Figures and Tables

**Table 1:** Worldwide prevalence of ocular infections and their causative agents.

Ocular Disease (Most Common)	Primary Bacterial Agents (~% of Isolates)	Typical Regions with Higher Reports	References
Acute bacterial conjunctivitis	*Staphylococcus aureus* (~20–30%), *Coagulase-negative Staphylococci* (~50%), *Haemophilus influenzae* (~10%)	Europe and North America (*Staphylococcus* dominant); Asia and Africa (higher *H. influenzae* in children)	[1,9]
Keratitis (corneal ulcer)	*Pseudomonas aeruginosa* (~30–40%), *Staphylococcus aureus* (~15%), *Streptococcus pneumoniae* (~10%)	Warm, humid climates (Southeast Asia, tropical regions) show more *P. aeruginosa*; temperate zones report more *Staphylococci*	[1,9,10]
Blepharitis	*Coagulase-negative Staphylococci* (~60%), *Staphylococcus aureus* (~20%)	Worldwide, slightly higher prevalence in Europe and North America	[1,9]
Endophthalmitis (postsurgical)	*Staphylococcus aureus* (~25%), CoNS (~25%), *Streptococcus* spp. (~15%)	Reported globally, higher Gram-positive rates in developed countries	[1,9]
Dacryocystitis	*Staphylococcus aureus* (~30%), *Streptococcus* spp. (~20%), *Pseudomonas* spp. (~10%)	More frequent in regions with limited access to ophthalmic care (Africa, some parts of Asia)	[1,9]

**Table 2:** Ocular pathogens and their clinical complications.

Pathogen Type	Common Species	Complications	References
Bacterial	*Staphylococcus aureus*, *Pseudomonas aeruginosa*	Contact lenses, MRSA, ESKAPE resistance	[18,31]
Fungal	*Fusarium*, *Aspergilli*, *Candida*	Trauma, agriculture sites, corneal transplant	[1,2]
Viral	Herpes Simplex Virus (HSV), Cytomegalovirus (CMV)	Neovascularization (VEGF), antiviral resistance	[27,32]

**Table 3:** Major genetic determinants of antimicrobial resistance in ocular pathogens.

Resistance Gene/Mechanism	Associated Pathogen(s)	Mechanism of Resistance	References
mecA	*Staphylococcus aureus*, CoNS	Production of altered penicillin-binding protein (PBP2a)	[18,45]
*gyrA*, *gyrB* mutations	*Pseudomonas aeruginosa*, *S. aureus*	Altered DNA gyrase target	[46]
*parC*, *parE* mutations	*P. aeruginosa*, CoNS	Altered topoisomerase IV	[44]
*qnrA*, *qnrB*, *qnrS*, *qnrVC1*	*P. aeruginosa* and Gram-negative isolates	Protection of DNA gyrase/topoisomerase	[18]
*erm* genes	*S. aureus*, *Streptococci*, CoNS	23S rRNA methylation	[46]
*msrA*, *mefA*	*Staphylococci*, *Streptococci*	Active drug efflux	[47]
blaTEM, blaSHV, blaCTX-M	*Enterobacterales*, Gram-negative ocular isolates	β-lactam hydrolysis	[44]
Carbapenemases (NDM, KPC, OXA)	Gram-negative pathogens	Carbapenem degradation	[18]
*aac(6′)-Ib*, *aph*, *ant* genes	Gram-negative bacteria, *Staphylococci*	Aminoglycoside modification	[46]
Mobile genetic elements (plasmids, integrons, transposons)	Multiple pathogens	Horizontal gene transfer	[47]
NDM, KPC, OXA-48-like	Gram-negative ocular pathogens	Carbapenem degradation	[14]
*aac(6′)-Ib*, *aph*, *ant* genes	Gram-negative bacteria, *Staphylococci*	Aminoglycoside modification	[29]
*VanA*, *VanB*	*Enterococcus* spp.	Altered peptidoglycan precursor causing vancomycin resistance	[29]
*tet(M)*, *tet(K)*, *tet(L)*	*S. aureus*, *Streptococci*, CoNS	Tetracycline ribosomal protection/efflux	[28]
*cat* genes	*Staphylococci*, Gram-negative isolates	Chloramphenicol acetylation and inactivation	[27]

**Table 4:** Current therapeutic approaches and clinical challenges in ocular infections.

Infection Type	Key Therapy	Major Challenges	Clinical Insights	References
Bacterial	Empiric therapy to culture-guided antibiotics.	Antimicrobial resistance, biofilms, persister cells, poor drug retention.	Rapid intervention, biofilms lead to treatment failure and relapse in the cornea.	[10,13,58,59,62,63,65,69]
Fungal	Natamycin, voriconazole, Penetrating keratoplasty therapy, Rose Bengal photodynamic therapy	Poor corneal penetration, delayed diagnosis, slow culture growth.	Severe infection, highly prone to surgical intervention.	[75,76,77,78]
Viral	Antivirals (acyclovir, valacyclovir), Human Papillomavirus (HPV), Cytomegalovirus (CMV), Herpes Simplex Virus (HSV)	Latency and recurrence	Requires long-term management and prophylaxis	[80,81,83,84]

## Data Availability

No new data were created or analyzed in this study. Data sharing is not applicable to this article.

## Abbreviations

The following abbreviations are used in this manuscript:

AMR	Antimicrobial Resistance
CMV	Cytomegalovirus
VEGF	Vascular Endothelial Growth Factor
MRSA	Methicillin-Resistant *Staphylococcus aureus*
ESKAPE	*Enterococcus faecium*, *Staphylococcus aureus*, *Klebsiella pneumoniae*, *Acinetobacter baumannii*, *Pseudomonas aeruginosa*, *Enterobacter* spp.
AMT	Amniotic Membrane Transplantation
EPS	Extracellular Polymeric Substance
HSV	Herpes Simplex Virus
rhNGF	Recombinant human nerve growth factor
EKC	Epidemic Keratoconjunctivitis
FDA	Food and Drug Administration
CRISPR-Cas9	Clustered Regularly Interspaced Short Palindromic Repeats–CRISPR-associated Protein 9
PCR	Polymerase Chain Reaction
mNGS	Metagenomic Next-Generation Sequencing
HPV	Human Papillomavirus
OSSN	Ocular Surface Squamous Neoplasia
GAG	Glycosaminoglycan
RNA	Ribonucleic Acid
*E6/E7 genes*	Oncogenic early genes of HPV
IFN-α2b	Interferon alpha-2b
MMC	Mitomycin-C
5-FU	5-Fluorouracil
*OprD*	Outer membrane protein D
QRDRs	Quinolone Resistance-Determining Regions
*gyrA*	DNA gyrase subunit A gyrB—DNA gyrase subunit B
*parC*	Part of Topoisomerase IV
*parE*	Part of Topoisomerase IV
PMQR	Plasmid-Mediated Quinolone Resistance
*qnrVC*	Quinolone Resistance Residual Vitreous Cortex
*qnrA*	Quinolone Resistance gene A
*qnrB*	Quinolone Resistance Gene B
*qnrS*	Quinolone Resistance gene S
SCCmec	Staphylococcal Cassette Chromosome mec
PBP2a	Penicillin-Binding Protein 2a
*msrA*	Methionine Sulfoxide Reductase
*mefA*	Macrolide Efflux A
*msr(D)*	macrolide resistance gene
MSCRAMMs	Microbial Surface Components Recognizing Adhesive Matrix Molecules.
AI	Artificial Intelligence
MDR	Multi-drug resistant
CoNS	Coagulase-negative Staphylococcus
QSAR	Quantitative structure–activity relationship
WHO	World Health Organization
GLASS	Global Antimicrobial Resistance and Use Surveillance System
